# Identification of human MutY homolog (hMYH) as a repair enzyme for 2-hydroxyadenine in DNA and detection of multiple forms of hMYH located in nuclei and mitochondria

**DOI:** 10.1093/nar/gkv264

**Published:** 2015-03-23

**Authors:** T. Ohtsubo, K. Nishioka, Y. Imaiso, S. Iwai, H. Shimokawa, H. Oda, T. Fujiwara, Y. Nakabeppu

*Nucleic Acids Res*. 2000, **28**:1355–64.

The authors wish to make the following corrections to their article. The results and conclusion of the article are not affected and remain valid.

The authors apologise to the readers for the inconvenience caused.

**Figure 7A**

**Figure 7.**
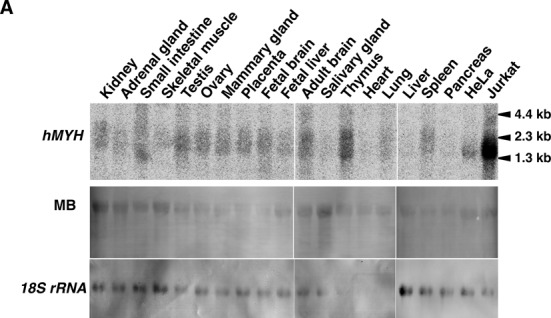
Expression of hMYH mRNAs in various human tissues and cell lines. (A) Northern blot analysis. Total RNAs (16 μg each) extracted from various human tissues, Jurkat and HeLa S3 cells were electrophoresed, transferred onto nitrocellulose membrane, and probed with ^32^P-labeled hMYH cDNA. The blots were stained with methylene blue (MB), and reprobed with 18S rRNA probe (*18S rRNA*).

Lane 7 should be Mammary gland NOT Placenta

Lane 8 should be Placenta NOT Mammary gland

Caption (new text underlined): Expression of hMYH mRNAs in various human tissues and cell lines. (A) Northern blot analysis. Total RNAs (16 μg each) extracted from various human tissues, Jurkat and HeLa S3 cells were electrophoresed, transferred onto nitrocellulose membrane, and probed with ^32^P-labeled hMYH cDNA. The blots were stained with methylene blue (MB), and reprobed with 18S rRNA probe (*18S rRNA*).

A new Figure 7 is shown below.

**Materials and Methods - Northern blot analysis**

The following sentence

“To confirm the amounts of RNA loaded, the blot was reprobed with a 1.0 kb *Eco*RI-*Bam*HI fragment of 18S rRNA gene prepared from pRγcHE (40), obtained from the Japanese Cancer Research Resources Bank.”

Has been corrected to

“To confirm the amounts of RNA loaded, the blot was stained with methylene blue, and reprobed with a digoxigenin-labeled 1015 bp fragment of 18S rRNA gene prepared by using PCR DIG probe synthesis kit (Roche Diagnostics, Mannheim, Germany), a forward primer (5′-TGCCAGCAGCCGCGGTAATTCC-3′), a reverse primer (5′-GAATAATTGCAATCCCCGATCC-3′), and a template plasmid pRγcHE (40), obtained from the Japanese Cancer Research Resources Bank.”

